# A case of neurolymphomatosis: A rare complication of diffuse large B‐cell lymphoma

**DOI:** 10.1002/jha2.141

**Published:** 2021-01-26

**Authors:** George Gabriel Bitar, Simon O'Connor, Ayoma D. Attygalle, Dima El‐Sharkawi, Sunil Iyengar, Bhupinder Sharma

**Affiliations:** ^1^ The Royal Marsden Hospital London UK; ^2^ The Institute of Cancer Research London UK

**FIGURE 1:**
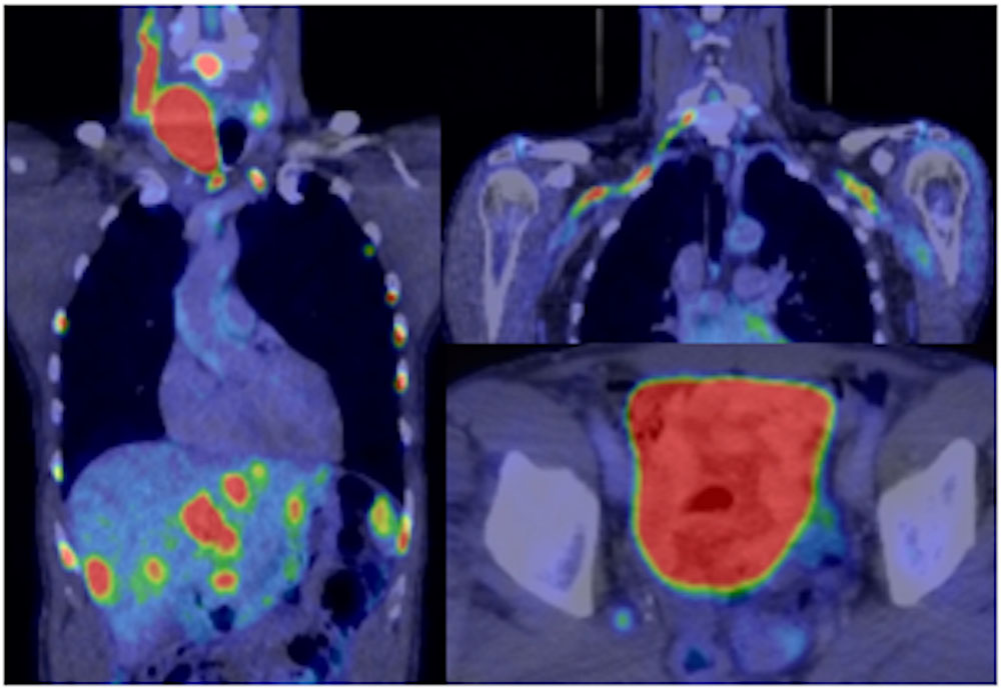
(Left) Coronal ^18^F‐FDG PET image demonstrating FDG avid right thyroid mass, cervical chain lymph nodes, liver and bony infiltration (ribs) consistent with stage IV lymphoma with three extra‐nodal sites of disease. (Top right) Coronal ^18^F‐FDG PET CT image demonstrating the ‘classic linear’ pattern of neurolymphomatosis with radiotracer uptake along the neurovascular bundles in the neck and axillae bilaterally. (Bottom right) Axial *^18^* F‐FDG PET‐CT image demonstrating a subtle focus of ^18^F‐FDG uptake along the right sciatic nerve, posterior to excreted radioactive tracer in the urinary bladder

**FIGURE 2:**
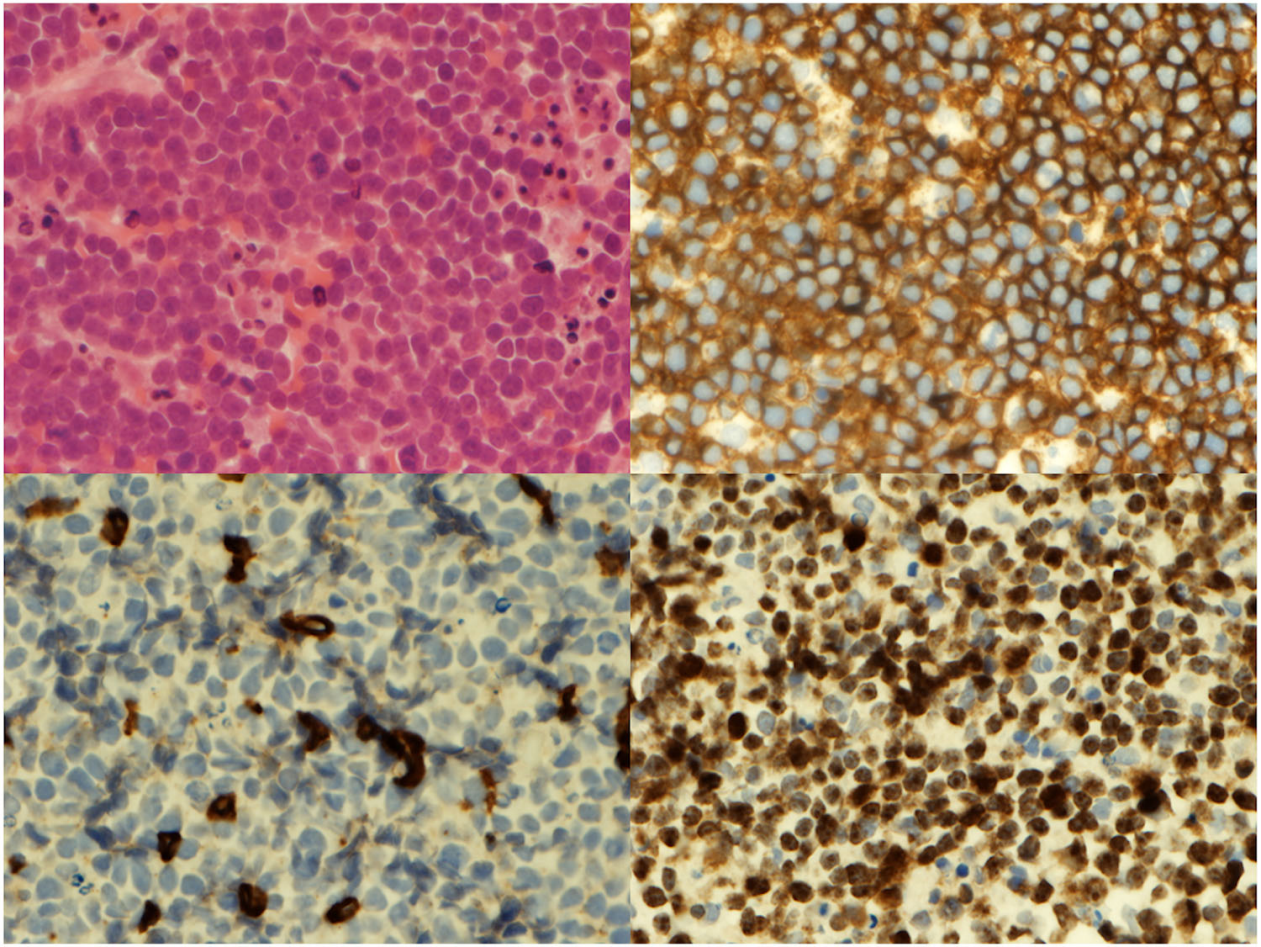
(Top left) H&E shows a diffuse population of large lymphoid cells. (Top right) CD20 immunostaining demonstrates strong positivity within the infiltrate. (Bottom left) CD3 highlights only occasional reactive T cells. (Bottom right) MIB confirms a high proliferative index of over 90%. All images are x60 objective

A 66‐year‐old man with no significant past medical history developed a swelling in the right side of his neck. The pre‐treatment ^18^F‐FDG PET‐CT (*as in figure* *1* *, left*) demonstrated a large FDG‐avid mass in the right thyroid lobe, enlarged right cervical chain lymph nodes and three sites of extra‐nodal involvement (liver, thyroid and bone marrow) consistent with a stage IV malignancy. An MRI head was normal. A right cervical lymph node core biopsy was replaced by a diffuse monomorphic population of medium‐sized to large lymphoid cells with scanty cytoplasm. Immunohistochemistry showed the abnormal cellular infiltrate to express the B‐cell antigens CD20 and CD79a, as well as BCL6, BCL2 and MYC with a high proliferative index of at least 90% (*as in figure* *2*). The histopathological findings were consistent with a diffuse large B‐cell lymphoma.

Central nervous system (CNS) prophylaxis was administered in the form of intrathecal methotrexate, and R‐CHOP was given. A good response to treatment ensued after three cycles of R‐CHOP, but it was decided to continue the intrathecal methotrexate in view of the high risk for CNS involvement.

Subsequently, the patient developed a painful, progressive neuropathy across his shoulder blades and spreading through both arms along with paraesthesia and pain in his right lower limb and a foot drop. The pain did not improve with amitriptyline or opiates.

A lumbar puncture revealed a clear cerebrospinal fluid. A repeat ^18^F‐FDG PET‐CT demonstrated complete resolution of the previously demonstrated disease however there was new FDG uptake at the right C6 neural foramen and extending inferolaterally along the subclavian and axillary vessels as well as adjacent to the left T1 neural foramen (*as in figure 1, top right*) consistent with peripheral neurolymphomatosis (NL). Further uptake was associated with the right L5 neural foramen and associated with the right sciatic nerve (*as in figure 1, bottom right*). A biopsy was deemed not to be feasible. The patient was commenced on dexamethasone and salvage chemotherapy with R‐ICE with an excellent clinical and radiological response following two cycles.

Peripheral NL is a rare complication of peripheral or cranial nerve root dysfunction, being most frequently secondary to infiltration by B‐cell non‐Hodgkin lymphoma [1], although it may occur secondary to NK‐cell, T‐cell lymphomas or leukaemias. Presenting symptoms are variable but include plexopathy, foot drop, cranial nerve palsies, and radiculopathy, and diagnosis (both radiological and histopathological) can be difficult. This case emphasises the crucial roles of PET‐CT in establishing the diagnosis (where the results of other diagnostic modalities, e.g., MRI, may be inconclusive) and for response assessment, whole body PET imaging being useful as different peripheral nerves may be affected in a relapsing/refractory pattern and demonstrates the ‘*classic linear’* ^18^F‐FDG uptake seen along the path of peripheral nerves/neurovascular bundles. Steroids alone provide only short‐term symptom control, and definitive treatment entails chemotherapy. Most of the limited number of reported cases of this rare entity have shown a poor clinical outcome.
